# Promising short-term results after anatomical total shoulder arthroplasty for glenohumeral osteoarthritis with a novel stemless humeral implant in combination with a pegged all-polyethylene bone ingrowth glenoid component

**DOI:** 10.1007/s00590-025-04405-2

**Published:** 2025-07-04

**Authors:** Spiros Tsamassiotis, Tomas Smith, Karen Rychlik, Roman F. Karkosch, Hauke Horstmann, Gunnar Jensen

**Affiliations:** 1 Department of Orthopaedic Surgery, Hannover Medical School, Diakovere Annastift, Hannover, Germany; 2 DePuy Synthes, Warsaw, United States

**Keywords:** Stemless shoulder arthroplasty, Arthritis shoulder, Anatomic shoulder arthroplasty, Clinical and radiological outcomes

## Abstract

**Purpose:**

Stemless anatomical shoulder arthroplasty offers numerous potential advantages over stemmed systems. The aim of this prospective study was to evaluate the clinical and radiological 2-year results of a novel metaphyseal anchored stemless shoulder system (Global Icon™ Stemless Shoulder System, DePuy Synthes, Warsaw, IN, USA).

**Methods:**

Thirty patients with primary osteoarthritis, 14 males and 6 females, were prospectively included in the study. Two-year follow-up data were analyzed for 20 patients. The average age and BMI were 63.4 years and 27.6 kg/m2. The adjusted Constant-Murley Score (aCS), Oxford Shoulder Score (OSS), and EQ-5D-5L were used as primary endpoints to reflect postoperative clinical improvement. Postoperative radiographs in two planes were analyzed for implant loosening and migration.

**Results:**

There was an overall clinical improvement 2 years postoperatively. The aCS increased from 58.3 ± 17.7 to 113.7 ± 15.6 points (p < 0.0001). The OSS score improved from 25.5 ± 6.1 to 43.9 ± 6.1 points (*p* < 0.0001). The EQ-5D-5L value score increased on average from 0.7 ± 0.2 to 0.9 ± 0.1 (*p* = 0.0004). There was no implant loosening or migration. No revisions were required.

**Conclusion:**

In summary, the examined prosthesis system achieved very good clinical and radiographic results 2 years postoperatively without implant failure. Shoulder function and quality of life improved significantly. These results are encouraging for prostheses with bone-preserving designs which rely on peripheral humeral fixation, though more long-term results are needed.

**Level of evidence:**

Level IV; Case Series; Treatment Study.

## Introduction

Total shoulder arthroplasty (TSA) has been continuously developed over the years to improve implant function and restoration of the patient's specific anatomy [1]. According to current data, stemless aTSA does not appear to be inferior to stemmed aTSA in terms of short- to medium-term results [1, 2]. It also has a comparable risk of complications, although the incidence of intraoperative fractures is lower with the stemless prosthesis [3]. Further, the stemless aTSA allows greater flexibility adapting to anatomical variants of the proximal humerus than a stemmed implant[4]. Potential advantages of a TSA include better preservation of the native bone, shorter operating time, reduced stress-shielding, blood loss, and shorter hospital stay for the patient. These benefits support the relevance of the new stemless implants [1, 2, 5].

The aim of this prospective study was, therefore, to evaluate the clinical and radiographic 2-year results of the Global Icon™ Stemless Shoulder System, since current clinical data regarding this implant are limited. There are numerous different stemless designs existing, which follow various fixation principles. Computational analysis of finite element models indicates that different designs of anatomical aTSA have a relevant influence on humeral bone adaption process, such as increased bone resorption and thus increased bone mass loss [6]. The main innovations of this stemless prosthesis design are fixation in the periphery of the proximal humerus, where bone density is higher, and a bone-preserving design with easy removal in the case of revision. Currently, only retrospectively collected clinical data are available for the medium-term results of this implant system.

We hypothesized that patients with primary osteoarthritis will experience a significant clinical benefit 2 years after implantation of the examined aTSA.

## Materials & methods

### Population

Between December 2017 and July 2019, 30 patients who met the inclusion criteria were prospectively included in the study. The inclusion criteria were defined as severely painful and disabling osteoarthritis or post-traumatic arthritis, a patient age between 21 and 80 years, an intact rotator cuff, sufficient bone quality for secure implant fixation, and the absence of a shoulder prosthesis on the contralateral side. 

The exclusion criteria included fractures of the proximal humerus and/or other previous operations that could compromise implant fixation, local and/or systemic infections, revision TSA, ongoing medical treatments that affect bone quality, pregnancy or breastfeeding, relevant allergies and severe mental illness, as well as alcohol and drug abuse.

All participants signed a written informed consent, and the study was approved by the local ethics committee.

During the inclusion period, 26 patients met the inclusion criteria. Among these, six were lost to follow-up, so that 20 patients completed the 2-year follow-up (follow-up rate: 77%). There were 14 male and 6 female patients. The average age was 63.4 years (47–80), and the average BMI was 27.6 kg/m2 (19.9–37.6). The indication for joint replacement was primary osteoarthritis in 19 cases and instability arthritis in one case.

Bone quality was assessed only intraoperatively using the Churchill thumb test [7].

### Surgical technique and implant design

The novel humeral fixation of the Global Icon™ consists of an anchor plate, which is designed for fixation in the peripheral bone area of the proximal humerus, where bone mass and density are higher than in the central bone [8] and a humeral head, which is connected to the anchor plate via a female Morse taper. Together with the anatomically designed sizes of the humeral head components, the patient`s individual anatomy can be reconstructed according to the best-fit circle principle to restore the individual offset, retroversion, and inclination. The four peripheral legs of the anchor plate have a grooved macrostructure and are T-shaped. This increases the fixation surface and implant stability.

All surgeries were performed by two experienced shoulder surgeons. The standard deltopectoral approach was chosen. The subscapularis tendon was detached using the peel-off technique [9]. A guided anatomical resection of the humeral head was performed orientated at the superior and posterior attachment point of the rotator cuff. The resection surface on the anatomical neck was orientated to the native inclination and retroversion using a control template. A target instrument corresponding to the native glenoid size was used to place a guide wire for the Peg Glenoid with a desired retroversion between 0 and 10°. For glenoids with Walch type B morphologies, high-sided reaming was conducted. Trans-osseous drill holes were created in the lesser tuberosity for refixation of the subscapularis tendon, and the tendon was carefully reconstructed using non-absorbable suture material.

### Rehabilitation protocol

The shoulder was immobilized in an abduction brace for 4 weeks. Patients received physiotherapy from the 1st postoperative day on with passive joint motion. External rotation was limited to half of the intraoperatively assessed external rotation capability for 6 weeks. Flexion and abduction were also limited to 90° for 6 weeks, and internal rotation was allowed in front of the body without resistance.

### Clinical and radiological outcome measures

The adjusted CS (aCS) according to Constant et al. [10], OSS, and EQ-5D-5L were assessed as primary endpoints at baseline and 24 months postoperatively. Intra- and postoperative complications were recorded.

**Fig. 1:**
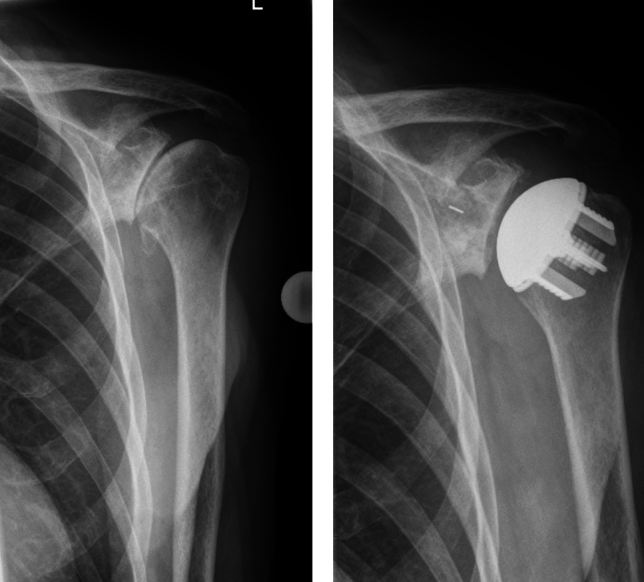
Preoperative shoulder true AP view (left) and after prosthesis implantation (right)

Radiographs were taken in two planes (true a.p. and axial) and assessed by an independent radiologist for radiolucencies, osteolysis, loosening, migration, fractures, device condition, glenohumeral subluxation, and subsidence according to a previously published method for the humeral component [11] (Fig. 1). The true a.p. plane was repeated if insufficient. Radiolucencies regarding the pegged glenoid component were assessed according to Lazarus et al. [12].

**Fig. 2:**
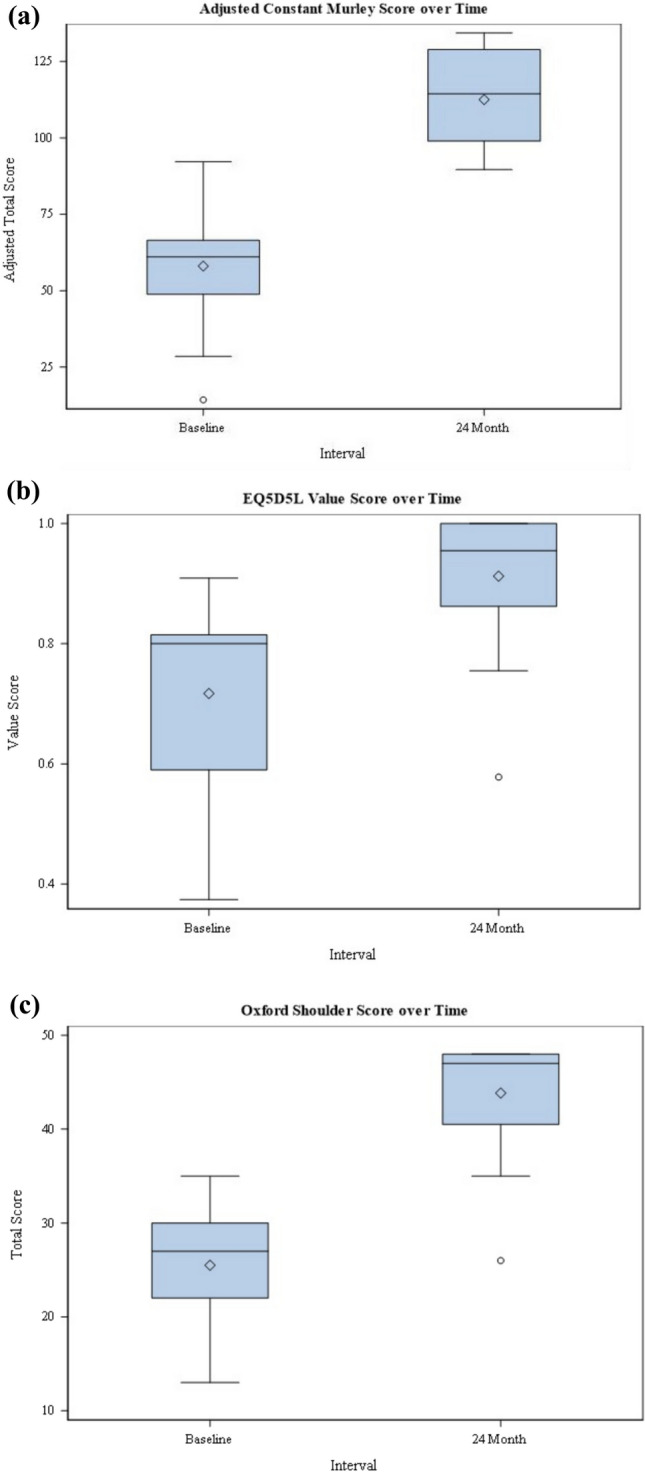
Box plots demonstrating the aCS (**A**), EQ-5D-5L (**B**), and the OSS (**C**) over time

Glenoid morphology was determined on preoperative two-dimensional cross-sectional imaging using the modified Walch classification, and glenoid retroversion was measured according to the Friedman method. Also based on cross-sectional imaging, fatty degeneration of the rotator cuff was assessed using the simplified Goutallier classification according to Fuchs and atrophy of the supraspinatus muscle according to Thomazeau (Fig. 2).

### Statistical analysis

For the primary endpoints aCS, OSS, and EQ-5D-5L, post hoc comparisons between the baseline values and the follow-up were performed via paired t-test and visualized as box plots. The significance level was set at *p* < 0.05. The above-mentioned variables and demographics, surgical characteristics, adverse events, radiographic data, glenoid morphology, glenoid retroversion, supraspinatus atrophy, and fatty degeneration of the rotator cuff were also analyzed descriptively. Type A and B glenoids, Thomazeau I and II, as well as the normal and moderate grades according to the simplified Goutallier classification were compared in relation to the score results and analyzed using an independent t-test. Fisher’s exact tests were used for tests of association for categorical classifications. Statistical analyses were performed using SAS (version 9.4).

## Results

### Clinical outcome

The aCS increased from 58.3 ± 17.7 preoperatively to 113.7 ± 15.6 postoperatively (p < 0.0001). The OSS also increased from 25.5 ± 6.1 points preoperatively to 43.9 ± 6.1 points postoperatively (*p* < 0.0001). The EQ-5D-5L value score increased from 0.7 ± 0.2 preoperatively to 0.9 ± 0.1 (*p* = 0.0004) (Fig. 1).

### Implant survivorship

Implant survivorship at 2-year follow-up was 100%. No revision was necessary, and no postoperative infections were recorded.

### Cross-sectional imaging results

Four glenoids were classified as A1 (20%), 3 as A2 (15%), 2 as B1 (10%), 5 as B2 (25%), 5 as B3 (25%), and 1 as D (5%). The mean glenoid retroversion was 11° ± 7.3° (mean ± SD) and ranged from − 4° to 21°. Fifteen subjects were classified as “normal” and 5 as “moderate” according to the Goutallier classification. Thirteen patients showed I° and 7 II° atrophy according to Thomazeau. Walch classification was dichotomized into A and B, and no significant associations were found regarding clinical and radiographic outcome.

### Radiographic results

A periprosthetic glenoid fracture was seen on the radiographs 3 months after surgery. A lesser tuberosity fracture occurred after a fall. In both patients, no intervention was necessary, and the fractures had no negative impact on the clinical and radiographic outcomes. Otherwise, no radiolucencies, osteolysis, migration, subsidence, or aseptic loosening were detected regarding the humeral component in the radiographic follow-up. The device condition was intact, and no humeral subluxation was found. Nine pegged glenoids were classified as grade 0 (45%), 10 as grade 1 (50%), and one pegged glenoid as grade 2 (5%) according to Lazarus. Lazarus grading scale was dichotomized into 0 and > 0 (or 1 and 2), and no significant associations were found regarding clinical and radiographic outcome.

## Discussion

The aim of this prospective study was to determine the clinical and radiological short-term results of the stemless Global Icon™ Shoulder System in combination with the Global® Anchor Peg Glenoid. A significant increase was observed in all scores 2 years postoperatively, both regarding the function of the operated shoulder and the quality of life. The EQ-5D-5L also showed a significant increase. Thus, our study was also able to demonstrate a short-term increase in quality of life after implantation of the prosthesis. Our implant-related complication rate (two patients) and inconspicuous radiological results are also in line with the data from the literature [13].

One retrospective study of 30 patients who underwent aTSA with the Global Icon™ system showed an average increase in OSS of 20.1 points. The authors reported no complications and normal radiographs. The glenoids of 29 patients were typed. Here, 37.93% of the patients had an A2 glenoid, 20.68% an A1, 17.24% a B2, 10.35% a B1, and 13.8% a type C glenoid [14]. Our collective did not include any C-glenoids but had a higher proportion of B-glenoids. The study does not clearly describe how the increased retroversion of the C-glenoids was managed intraoperatively. However, the distribution of glenoid types in our study is consistent with the literature [15].

With an average retroversion of 11° (− 4 to 21°) in our study, the desired postoperative retroversion between 0 and 10° was achieved by eccentric reaming. However, our investigated population shows a high number of B-glenoids (60%). It has been shown that higher retroversion angles and humeral subluxation significantly increase the risk of complications [16]. Therefore, B2 glenoids in particular have been associated with a lower implant survival rate, mainly due to early glenoid loosening in the mid- to long-term follow-up. More recent registry data, on the other hand, show that preoperative glenoid morphology is not a risk factor for revision [17]. Although the study population was rather small to evaluate this aspect, there was no significant difference between A and B glenoids in our study population. This has also been shown in other studies [17, 18]. Glenoid radiolucency rates of up to 96% have been described for aTSA [19]. In a retrospective multicenter study with an average follow-up of 28 months using a hybrid all-polyethylene glenoid, 49.5% of 109 patients were classified as Lazarus grade 0, 39.4% as grade 1 and 11% as Lazarus grade 2 [20]. Another study was able to demonstrate grades 0–1 radiolucencies according to Lazarus in 81.6% of patients in the short- to mid-term follow-up of a hybrid all-polyethylene glenoid [21]. The radiographic results of our study are similar to the relevant literature. In our study, no significant influence of preoperative glenoid morphology on the outcome could be demonstrated. However, our study was underpowered in this regard, and the follow-up was short.

In a systematic review article of stemless TSA, humeral radiolucencies were reported in 7.1%, stress-shielding in 7.7%, and migration in 0.7%. At an average follow-up of 2.5 years, 0.9% progressive osteolysis was reported [22]. However, apart from three cases with rheumatoid arthritis, no correlation between the radiographic and clinical results could be demonstrated. A recent prospective multicenter study of 61 patients who received a stemless aTSA showed significant improvement of aCS, OSS, and EQ-5D-5L without radiological abnormalities at a 2-year follow-up, as in our study [23]. Another review of stemless TSA systems showed a complication rate of 0–7.9% at an average follow-up of 26 months for the humeral components. However, it appears that some prosthetic systems are more associated with radiologic changes than others, perhaps due to implant design, but all without clinical relevance [24]. In our study, the survival rate was 100%. In a retrospective series of 100 patients with a follow-up of 2 years, five revisions occurred. None were implant-related, and four were due to secondary cuff failure. A radiolucent line was seen in only one patient, and no infection or loosening was detected [25].

Australian registry data over a 7-year period showed equal revision rates for stemless and stemmed aTSA. [17]. Another study comparing stemless versus stemmed aTSA, based on the New Zealand registry data with revision after 7 years as the primary outcome, found no significant difference in the revision rate. In contrast with the stemmed group, there were no loosening or intraoperative fractures in the stemless group. The stemless group performed significantly better in the OSS 6 months postoperatively than the stemmed group [13]. Parameters of stemless implants that have not been well investigated so far, such as proximal bone response, mechanical engagement, and different design concepts also need to be examined in mid- and long-term studies. Computational analysis showing different bone responses depending on prosthesis designs and the relatively high complication rates of over 10% in some studies also underline the need for further research [6, 26]. The latest EFORT review concludes that stemless implants have the same clinical outcomes, complication, and revision rates as stemmed implants, with fewer intraoperative fractures and peri-implant-associated radiological abnormalities. Among the individual stemless implants though, significant differences were found regarding clinical outcome, complication, and revision rates, with great study heterogeneity and a low level of evidence. In addition, most of the data relate to short-term follow-ups and by no means have the majority of existing implant systems been published. Here too, the authors conclude that well-designed long-term studies are essential [22].

As far as we know, the implant used in this study has not been examined in any review article so far. In addition, this is the first study to prospectively collect 2-year results of this implant system in combination with an all-polyethylene pegged bone ingrowth glenoid component. Thus, our study provides valuable insights into this new implant device and demonstrates the effectiveness and safety of the prosthesis design in the short-term follow-up. A further strength of the study is the carefully selected patient collective. There was only one secondary instability osteoarthritis with otherwise exclusively primary osteoarthritis. In addition, 15 patients had normal muscle status according to the simplified Goutallier classification, and the remaining patients had moderate fatty degeneration. There was also no high-grade atrophy of the rotator cuff, as 13 patients were staged Thomazeau stage I and the remaining Thomazeau stage II.

Weaknesses of this study are the small number of patients and the short follow-up. Another limitation of the study is that glenoid version was measured using two-dimensional cross-sectional imaging, rather than three-dimensional cross-sectional imaging, which provides more accurate measurements. Furthermore, no objective measurement of bone quality was obtained, e.g., by DXA. In addition, there was no comparison group and, therefore, no randomization.

## Conclusions

Our study demonstrates very good clinical outcomes for the novel humeral implant system examined, which were not only significant but also clinically relevant, at 2-year follow-up. The overall complication rate was low. Radiographic outcomes showed an implant survivorship of 100% in absence of further radiological findings like aseptic loosening.

## Data Availability

No datasets were generated or analysed during the current study.
